# HIV-related knowledge, attitude and practices of healthy adults in Cross River State Nigeria: a population based-survey

**DOI:** 10.11604/pamj.2017.27.170.12082

**Published:** 2017-07-04

**Authors:** Uchenna Okonkwo, Soter Ameh, Akaninyene Otu, Henry Okpara

**Affiliations:** 1 Gastroenterology/Hepatology Unit, Department of Internal Medicine, University of Calabar Teaching Hospital, Calabar, Nigeria; 2 Department of Community Medicine, University of Calabar Teaching Hospital, Calabar, Nigeria; 3 Infectious Disease Unit, Department of Internal Medicine, University of Calabar Teaching Hospital, Calabar, Nigeria; 4 Department of Chemical pathology, University of Calabar Teaching Hospital, Calabar, Nigeria

**Keywords:** HIV, knowledge, attitude and practice, Nigeria

## Abstract

**Introduction:**

Human Immunodeficiency Virus (HIV) remains a global health problem disproportionately distributed across Nigeria. Cross river state (CRS), a tourist state, located in the Niger delta, has one of the highest prevalence rates. There is evidence that poor knowledge and stigmatization are obstacles to achieving universal access to HIV prevention programs. The objective of this study was to determine the Knowledge, Attitude and Practice (KAP) of HIV among adults resident in CRS, Nigeria.

**Methods:**

A cross sectional descriptive survey design was employed. A total of 1,620 healthy adults were recruited. KAP towards HIV was assessed using a structured pre-tested questionnaire. Categorical variables were described as frequencies and continuous variables as median and interquartile range. Kruskal-Wallis test was used to determine relationship between variables and median KAP scores. P value < 0.05 was considered significant. All analyses were performed using Stata 12 statistical package.

**Results:**

A total of 1,465 respondents completed the questionnaire correctly giving a response rate of 91%. The M: F ratio was 1:1.8. The median age was 38 years. Majority was married and had formal education. Knowledge of HIV and common routes of transmission was high (>80%). However, misconception that HIV can be transmitted through hugging, hand shake, mosquito bites and witch craft was also common (> 60%). The overall attitude and practice towards persons living with HIV infection was poor.

**Conclusion:**

This study showed misconceptions in the knowledge and consequences of HIV infection which is associated with negative attitude towards persons living with HIV.

## Introduction

Human Immunodeficiency Virus (HIV) is a global epidemic currently affecting approximately 37 million persons [1]. Since the beginning of the epidemic in the 80s, more than 70million people have been affected and 35million of them have died [2]. Although tremendous progress has been made in combating the pandemic globally with a 43% reduction in the annual Acquired immunodeficiency syndrome (AIDS) related mortality, huge challenges still lie ahead [1]. The burden of HIV/AIDS is not evenly distributed across or within countries and regions. Sub-Saharan Africa is the region of the world most severely affected by HIV/AIDS accounting for 70% of people living with HIV/AIDS (PLWHA) worldwide. This translates to almost 1 in every 25 adults (4.4%) living with HIV in the region [2]. Nigeria being the most populous country in sub-Saharan Africa is home to 3.5 million people with HIV infection accounting for 9.4% of the global population of PLWHA. Although, the adult HIV prevalence in Nigeria is relatively low at 3.1% compared to other African countries such as Zambia, South Africa and Chad with two digit prevalence rates, an estimated 250,000 new infections and 180,000 AIDS related deaths were recorded in Nigeria in 2015 with most of them in persons aged 15-49 years [3]. In spite of these disturbing figures, the National HIV/AIDS and Reproductive Health survey of 2012 showed that uptake of HIV testing is low with just 23% of males and 29% of females knowing their status [4]. In addition; only 24% of adults who were HIV positive were receiving anti-retroviral drugs. Thus, over 70% of infected persons remain untreated and serve as sources of new infection. Cross River State (CRS) located in the south-south geographical region of Nigeria is known for it's many international festivals of which the most notable is the annual carnival float which attracts tourists from all over the world. CRS had one of the highest prevalence rates of HIV in the country at the outset of the epidemic. However, with concerted efforts by the government and its agencies, the prevalence of HIV had dropped from 12% in 2003 to 6.9% in 2014 [5]. Nonetheless, this is still higher than the national average of 3.1%. Some of the drivers of this epidemic in the State include high risk sexual behavior and low risk perception of HIV and its consequences [6]. The Cross River State Agency for the Control of AIDS (CRSACA) reported that 75% of the population in the State do not perceive themselves as being personally at risk of HIV infection [7]. Unprotected heterosexual sex is the predominant mode of HIV transmission in Nigeria accounting for about 80% of new HIV infections [8]. Majority of the rest occurs among minority groups such as sex workers, men who have sex with men and intravenous drug users. Although these groups make up less than 2% of the Nigerian population, they account for around 23% of new HIV infections [9, 10]. Unsafe injection practices and mother-to-child transmission are other significant modes of transmission [11, 12]. Transfusion of unscreened blood or blood products was reported not to be a major source of new infection [13]. Studies in Nigeria among segments of the general population have shown that while knowledge of the nature of HIV is high, practice of unsafe sexual behavior is common and is associated with low knowledge of modes of transmission and risk perception [14–16]. The Nigerian Ministry of Health in 2013, reported that only 24% of young people could correctly identify ways to prevent sexual transmission of HIV, and reject common myths [4]. Moreover, the attitude towards PLWHAs is generally negative even among healthcare workers [16, 17–19]. The United Nations as part of its Sustainable Development Goals, committed to ending the HIV epidemic by 2030 [20]. For Nigeria to achieve this mandate, KAP studies are necessary to develop suitable health promotion interventions to facilitate health behavior change. It is hoped that this study will add to available knowledge and help promote already existing efforts in the fight against HIV/AIDS. The aim of this study was to determine the Knowledge, Attitude and Practice of HIV among adults resident of CRS, Nigeria.

## Methods

**Study setting:** CRS is located in the Niger delta region of Nigeria. There are 18 local government areas (LGAs) in CRS divided into three senatorial districts: Southern, Central and Northern senatorial districts. There are 6 LGAs per senatorial district and these are: Akamkpa, Akpabuyo, Bakassi, Calabar Municipal, Calabar South and Odukpani in the southern district, Abi, Biase, Boki, Etung, Ikom and Yakuur in the central district, Obanliku, Obubra, Obudu, Ogoja, Bekwarra, and Yala in the northern district. Each LGA comprises 10 wards.

**Study design:** This was a cross sectional study conducted between March and September, 2015.

**Study population:** The study population consisted of adult men and women (aged 18years and above) resident in CRS.

**Inclusion and exclusion criteria:** Individuals residing in the selected LGAs who were aged 18 years and above and provided written informed consent were included in the study while those not meeting these criteria were excluded.

**Sample size estimation:** A prevalence of 12%, confidence interval of 95% and precision degree of 5% was substituted into the Leslie and Kish formula, n = z^2^ p (1-p)/d^2^ to determine the sample size. An attrition rate of 10% was added and rounded up to 180 persons per LGA. These number of persons were recruited in the nine LGA studied to give a total of 1620.

**Sampling technique:** A stratified random sampling technique was employed. Three LGAs were randomly selected by balloting from each of the three senatorial districts. There were Calabar south, Akamkpa and Akpabuyo from the south, Abi, Ikom and Yakurr from the central, Ogoja, Obudu and Yala from the north. In each of the LGAs, three wards were randomly selected except in one LGA where 5 wards were selected for ease of access. A total of 29 wards in 9 LGAs in 3 senatorial districts were sampled.

**Data collection:** A structured 35 item questionnaire divided into three sections (excluding the demographic section) was used for data collection. It comprised of 27 questions on knowledge of HIV, 5 questions on attitude and 3 questions on practices towards PLWHA. The questionnaire was developed following extensive literature review and pre-tested for reliability and validity.

**Statistical analysis:** Data was entered into Microsoft Excel 2013 and imported into Stata 12.0 (College Station, TX, USA) for statistical analyses. It was declared as survey data by using the “svyset” Stata syntax before commencement of statistical analysis in order to minimize underestimation of the standard error arising from the survey design of the study. Continuous variables were reported in median and interquartile range because of the skewed nature of the data which was determined by Kolmogorov-Smimov test, whereas categorical variables were presented in frequencies (n) and relative frequencies (%). For knowledge and practice questions, a score of 1 was given to “yes” correct responses while a score of 0 was given to “no” incorrect responses. On the other hand, a score of 1 was assigned to “No” correct responses, whereas a score of 0 was assigned to “yes” incorrect responses (e.g. can HIV be transmitted through witchcraft?). For attitude questions, a score of 1 was assigned to “disagree” responses while a score of 0 was given to “agree” responses (e.g. non-infected persons should not work in the same office as HIV patients). The range of scores for knowledge, attitude and practice were 0-27, 0-5 and 0-3, respectively. The KAP scores were categorized as follows; for knowledge, score of 0-10 = knowledge is minimum, 11-20 = knowledge is adequate, >20 =knowledge is very good. Attitude score of 0-2 = attitude is negative, 3-4 = attitude is fairly positive, >4 = attitude is very positive. Practice score of 0-2 = practice is poor, >2 = practice is good. Spearman correlation was used to assess the relationship between knowledge, attitude and practice HIV scores. All statistical tests were undertaken at 5% significance level.

**Ethical consideration:** Ethical approval was obtained from the Cross River State Health Research Ethics Committee (CRS-HREC) with reference number RP/REC/2015/281. Respondents’ anonymity and confidentiality was maintained throughout the course of the study.

## Results

A total of 1,465 respondents completed the questionnaire correctly giving a response rate of 91%. There were 928 females (63.3%) and 517 males (35.3%). Twenty persons (1.36%) did not indicate their gender. The median age of the study participants was 38years. Sixty percent were married and 78% had formal education mostly tertiary education. There were 520 (35.4%), 517 (35.2%) and 428 (29.2%) respondents from the southern, northern and central senatorial districts respectively. The majority of the respondents (92%) had heard of HIV but only 52% were aware that it causes chronic infection. The knowledge of the modes of transmission of HIV was also high at 90% for sexual route, 88%, 87%, and 81% for blood transfusion, sharps and mother-to-child transmission respectively. Nonetheless, 78%, 67% and 62.5% responded erroneously that HIV could be transmitted through hugging, handshake, mosquito bites and witchcraft. Concerning knowledge of HIV prevention and control, majority believed that prevention of mother-to-child transmission by treating positive mothers was effective while only 13% agreed that screening family members of HIV positive persons was of any benefit. Responses to other knowledge questions are shown in Table 1. Our study showed that majority of the respondents agreed to the negative attitude and practice statements of not living (73%), eating (71.3%), working (74%) or marrying (77%) a person with a positive HIV status. Only 35% perceived themselves at risk of HIV infection. Median scores of the knowledge of HIV varied significantly with age, level of education and marital status (p < 0.05). Older persons, those without formal education, the widowed and divorced tended to have lower scores. Attitude scores also showed significant variation with age, level of education and senatorial districts with young persons, those without formal education and persons resident in the southern senatorial district having lower scores (p < 0.05). Practice scores did not show any significant variation with social demographic characteristics (Table 2). Knowledge of HIV infection was significantly correlated with attitude towards PLWHA and also with practices towards HIV prevention in all the senatorial districts (p < 0.001) respectively.

**Table 1:** Knowledge of the nature, prevention and transmission of HIV (n=1,465)

HIV knowledge items	Yes N (%)	Non N(%)
Knowledge of HIV		
Ever heard of HIV?	1,342 (91.6)	123 (8.4)
Can you tell a HIV- infected person from his/her appearance?	485 (33.1)	980 (66.9)
Does HIV cause life-long infection?	759 (51.8)	706 (48.2)
Is HIV more easily transmitted than Hepatitis B virus?	620 (42.3)	845 (57.7)
**Knowledge of HIV transmission**		
Can HIV be transmitted through		
Blood transfusion?	1,289 (88.0)	176(12.0)
Sexual intercourse?	1,321 (90.2)	141 (9.8)
Breastfeeding?	1,180 (80.5)	285 (19.5)
From infected mother to unborn child?	1,164 (79.5)	301 (20.5)
Sharing razor blade, nail cutter, clipper?	1,283 (87.6)	182 (12.4)
Sharing toothbrush with infected persons?	1,138 (77.7)	327 (22.3)
Scarification marks and tattoos?	1,062 (72.5)	403 (27.5)
Male or female traditional circumcision?	1,078 (73.6)	387 (26.4)
*Sharing food with HIV infected person?	1,043 (71.2)	422 (28.8)
* Eating food cooked by HIV infected person?	1,137 (77.6)	328 (22.4)
* Hugging?	1,144 (78.1)	321 (21.9)
Kissing?	724 (49.4)	741 (50.6)
* Hand shaking?	1,154 (78.8)	311 (21.2)
*Mosquito bites?	982 (67.0)	483 (33.0)
*Witchcraft?	916 (62.5)	549 (37.5)
**Knowledge of HIV prevention and control**		
Can HIV be prevented;		
* Through vaccination?	497 (33.9)	968 (66.1)
Screening of pregnant women?	1,188(81.1)	277 (18.9)
If HIV positive pregnant women receive treatment?	1,090 (74.4)	375 (25.6)
*If infants born to HIV positive mothers receive vaccination and immunoglobulin at birth?	945 (64.5)	520 (35.5)
If HIV positive mothers do not breastfeed their babies?	874 (59.7)	591 (40.3)
If family members of HIV patients are screened for HIV?	194 (13.2)	1,271 (86.8)
Is there a medical treatment for HIV?	992 (67.7)	473 (32.3)
*Is there a possibility of a cure of HIV?	638 (43.5)	827 (56.5)

* A score of 1 was given to “NO” responses while a score of 0 was given to “YES” responses

**Table 2:** Comparison of demographic characteristics and median KAP HIV scores

Variable	N (1,465)	Knowledge score Median (IQR)	P-value	Attitude score Median (IQR)	P-value	Practice score Median (IQR)	P-value
**Age group (years)**			0.003		<0.001		0.412
18-27	373	19 (15 - 22)		4 (1 - 5)		2 (1 - 2)	
28-37	315	20 (16 - 22)		5 (2 - 5)		2 (1 - 2)	
38-47	318	20 (16 - 22)		5 (2 - 5)		2 (1 - 2)	
48-57	216	20 (16.5 - 21)		4 (4 - 5)		2 (1 - 2)	
>57	168	18.5 (14 - 21)		5 (1.5 - 5)		2 (1 - 2)	
Missing	75	19 (16 - 22)		5 (1.5 - 5)		2 (1 - 2)	
**Sex**			0.882		0.752		0.149
Female	928	20 (16 - 22)		5 (2 - 5)		2 (1 - 2)	
Male	517	20 (16 - 22)		5 (2 - 5)		2 (1 - 2)	
Missing	20	18 (13.5 - 21.5)		5 (1.5 - 5)		2 (1 - 2)	
**Education**			0.0001		<0.001		0.213
None Primary	131 278	17 (11 - 20) 19 (14 - 21)		4 (0 - 4) 5 (1 - 5)		2 (1 - 2) 2 (1 - 2)	
Secondary	406	19(15-21)		4(2-5)		2(1-2)	
Tertiary	460	21 (18 - 22)		5 (3 - 5)		2 (1 - 2)	
Missing	190	20 (16 - 23)		5 (3 - 5)		2 (1 - 2)	
**Marital status**			0.029		0.235		0.436
Single	387	20 (16 - 23)		5 (2 - 5)		2 (1 - 2)	
Married	886	20 (16 - 22)		5 (2 - 5)		2 (1 - 2)	
Divorced	31	18 (12 - 21)		3 (0 - 5)		2 (1 - 2)	
Widowed	76	19(14.5-21)		5(1.5-5)		2(1-2)	
Missing	85	20(17-22)		5(4-5)		2(1-2)	
**Senatorial district**			0.002		<0.001		0.558
Southern	520	20(16-22)		4(1-5)		2(1-2)	
Central	428	20(17-22)		5(3-5)		2 (1 - 2)	
Northern	517	20(16-22)		5(2-5)		2(1-2)	

* Kruskal-Wallis test was used to detect a significant difference association of socio-demographic variables with the median KAP HIV scores

## Discussion

HIV remains a major public health problem in CRS and indeed Nigeria in spite of the concerted efforts by government to curb it over the last decade. Our study showed a good knowledge of HIV amongst residents of the three senatorial districts in Cross river state. While knowledge of the common routes of HIV transmission such as sexual intercourse, blood transfusion , sharing of sharps, scarification and tattooing was high, misconceptions that HIV can be transmitted through hugging, hand shake, mosquito bites and witch craft was also common. This finding is similar to what was reported in Benin city over a decade ago amongst civil servants and more recently among young persons in CRS and other developing countries such as Ghana, Lao Peoples Democratic Republic, and Afghanistan [14, 21–24]. In 2014, the Nigerian Bureau of Statistics reported that only 32% of the adult population in Nigeria had comprehensive knowledge of HIV transmission and prevention [25]. This is an indication of not only the need for more information and education of the general public concerning transmission of HIV but more importantly, the need to tailor the contents of such educational programs to address gaps in knowledge. The misconceptions surrounding the transmission of HIV are likely to negatively influence people's attitudes and behavior towards PLWHA and promoting stigma. Age varied significantly with knowledge with those aged 18-27years and above 57 years having lower scores. Persons in the former age group are more likely to be single while those in the later are more likely to have lost their partners considering the average life span of Nigerians is estimated at 52 years [25]. Nigeria remains a very moralistic society where sexual relationships receive societal approval only in the context of marriage and for procreation. This could affect the freedom of persons outside of marriage especially the younger age group to openly seek information about HIV against societal expectations [26]. Although education on HIV and related sexually transmitted diseases was recently incorporated in the Nigerian curriculum for primary and secondary schools, lack of adequately qualified teachers and counselors is a major challenge [27].

A negative attitude towards PLWHA was evident in the younger age group, persons with none or moderate education and those from the southern senatorial districts. The finding of a negative attitude towards PLWHA among the younger age group and people with low levels of education is not peculiar to our study. Similar results were reported in studies amongst youths in Nigeria and Tanzania [28, 29]. This may be due to fear of contracting the infection and stigmatization associated with being HIV positive. Ignorance, anxiety and fear of stigmatization have been identified as major barriers to the acquisition of accurate information on HIV and promote denial [30]. These concerns need to be addressed urgently to improve the acceptance of PLWHA in our communities and promote voluntary counseling and testing for HIV. The southern senatorial district is an urban area compared to the central and northern senatorial districts. The lower attitude scores in the southern district may probably be a reflection of the false impression on modes of transmission of HIV virus.

## Conclusion

This study shows that although the awareness of HIV and its major routes of transmission are high in CRS, there are gaps in the knowledge of the consequences of HIV and misconceptions about modes of transmission. There is need to develop community based inter-generational education programs to address barriers in communication and discriminatory attitudes.

### What is known about this topic

- HIV remains a disease of public health importance of global proportion despite concerted effort to combat it;
- The burden of HIV is high in Nigeria and is not evenly distributed within regions in the country;
- Uptake of HIV screening is low in Nigeria and it has been attributed to inadequate knowledge and low risk perception of HIV amongst the populace.

### What this study adds

- The study provides a population-based insight on the knowledge of HIV in Nigeria. Such community studies are uncommon in Nigeria;
- The study shows that misconceptions about the modes of transmission and low risk perception of HIV still exists even among educated persons in rural and urban areas of Cross River State, Nigeria;
- The study shows a significant correlation between knowledge, attitude and practice scores. It could be inferred that the high prevalence of negative and discriminatory attitude towards PLWHA is attributable to the misconstrued knowledge of the modes of transmission of HIV.

## Competing interests

The authors declare no competing interest.
